# *Pseudomonas syringae* pv. *syringae* Associated With Mango Trees, a Particular Pathogen Within the “Hodgepodge” of the *Pseudomonas syringae* Complex

**DOI:** 10.3389/fpls.2019.00570

**Published:** 2019-05-08

**Authors:** José A. Gutiérrez-Barranquero, Francisco M. Cazorla, Antonio de Vicente

**Affiliations:** Departamento de Microbiología, Facultad de Ciencias, Universidad de Málaga, Instituto de Hortofruticultura Subtropical y Mediterránea “La Mayora” (IHSM-UMA-CSIC), Málaga, Spain

**Keywords:** *Pseudomonas syringae* pv. *syringae*, mango tree, epiphytic fitness, virulence strategies, mangotoxin, pPT23A family plasmid, ultraviolet radiation and copper resistance

## Abstract

The *Pseudomonas syringae* complex comprises different genetic groups that include strains from both agricultural and environmental habitats. This complex group has been used for decades as a “hodgepodge,” including many taxonomically related species. More than 60 pathovars of *P. syringae* have been described based on distinct host ranges and disease symptoms they cause. These pathovars cause disease relying on an array of virulence mechanisms. However, *P. syringae* pv. *syringae* (Pss) is the most polyphagous bacterium in the *P. syringae* complex, based on its wide host range, that primarily affects woody and herbaceous host plants. In early 1990s, bacterial apical necrosis (BAN) of mango trees, a critical disease elicited by Pss in Southern Spain was described for the first time. Pss exhibits important epiphytic traits and virulence factors, which may promote its survival and pathogenicity in mango trees and in other plant hosts. Over more than two decades, Pss strains isolated from mango trees have been comprehensively investigated to elucidate the mechanisms that governs their epiphytic and pathogenic lifestyles. In particular, the vast majority of Pss strains isolated from mango trees produce an antimetabolite toxin, called mangotoxin, whose leading role in virulence has been clearly demonstrated. Moreover, phenotypic, genetic and phylogenetic approaches support that Pss strains producers of BAN symptoms on mango trees all belong to a single phylotype within phylogroup 2, are adapted to the mango host, and produce mangotoxin. Remarkably, a genome sequencing project of the Pss model strain UMAF0158 revealed the presence of other factors that may play major roles in its different lifestyles, such as the presence of two different type III secretion systems, two type VI secretion systems and an operon for cellulose biosynthesis. The role of cellulose in increasing mango leaf colonization and biofilm formation, and impairing virulence of Pss, suggests that cellulose may play a pivotal role with regards to the balance of its different lifestyles. In addition, 62-kb plasmids belonging to the pPT23A-family of plasmids (PFPs) have been strongly associated with Pss strains that inhabit mango trees. Further, complete sequence and comparative genomic analyses revealed major roles of PFPs in detoxification of copper compounds and ultraviolet radiation resistance, both improving the epiphytic lifestyle of Pss on mango surfaces. Hence, in this review we summarize the research that has been conducted on Pss by our research group to elucidate the molecular mechanisms that underpin the epiphytic and pathogenic lifestyle on mango trees. Finally, future directions in this particular plant–pathogen story are discussed.

## *Pseudomonas syringae* pv. *syringae* Strains Isolated From Mango Trees Belong to a Single Phylotype and Have Features Distinguishing Them From the Rest of the *Pseudomonas syringae* Complex

*Pseudomonas syringae* complex has been traditionally used as a taxonomic hodgepodge that currently includes 15 recognized bacterial species and more than 60 different pathovars of the *sensu stricto* species *P. syringae* (Gomila et al., 2017). The taxonomy of the *P. syringae* complex has been widely discussed over the last 40 years, yet still remains a controversial group. The classification of this group is defined based on host range and symptomatology, dividing *P. syringae* species into pathogenic varieties known as pathovars (Dye et al., 1980; Young, 2010). The pathovar-based classification is widely accepted even today, but does not reveal the genetic relationships between pathovars. Initial genomic studies were based on DNA-DNA hybridization methods (Palleroni et al., 1972; Pecknold and Grogan, 1973; Denny et al., 1988; Gardan et al., 1992; Janse et al., 1996). Gardan et al. (1999) described nine discrete genomospecies classification groups that have been widely accepted until recently. Phylogenetic approaches based on multilocus sequence typing analysis (MLST) have had a significant impact on *P. syringae* classification (Sarkar and Guttman, 2004; Hwang et al., 2005; Almeida et al., 2010; Bull et al., 2011; Berge et al., 2014). Although the classification proposed by Berge et al. (2014) is generally accepted, a recent study using comparative genomics of the whole genome sequences of this species proposed the delineation of phylogenomic *P. syringae* complex and confirmed, as one might expect, that a high proportion of strains were misclassified (Gomila et al., 2017). Significantly, different *P. syringae* strains isolated from different sources (i.e., snow, irrigation water, and a diseased crop) have been identified as belonging to the same evolutionary lineage (Monteil et al., 2016). This fact suggests that the evolutionary history of the plant pathogen *P. syringae* is linked to the water cycle, which promoted the colonization of agricultural and non-agricultural habitats (Morris et al., 2008).

*Pseudomonas syringae* species possess a great diversity of virulence factors, such as a type III secretion system (T3SS) and its effector repertoires, toxic compounds, exopolysaccharides, ice nucleation activity, cell-wall-degrading enzymes and plant hormones, that make it the model phytopathogenic bacterium for understanding plant–pathogen interactions. Additionally, adaptation mechanisms to its plant hosts and microbial evolution have more recently become of great interest to many research groups (Xin et al., 2018). In particular, *Pseudomonas syringae* pv. *syringae* (Pss), has been described as the most polyphagous bacterium into the *P. syringae* complex due to its broad host range (Kennelly et al., 2007). Pss strains isolated from mango trees were identified as the causative agent of bacterial apical necrosis (BAN) disease of mango trees, which is the most limiting factor for mango crop in the Mediterranean region (Cazorla et al., 1998). A novel antimetabolite toxin called “mangotoxin” was reported to be intimately associated with all Pss strains isolated from mango trees, and with a few Pss strains from other hosts (Arrebola et al., 2003). The presence of different variants of copper resistance genes, as well as ultraviolet resistance determinants, were found to be associated with 62-kb plasmids belonging to the pPT23A family plasmids (PFPs) (Cazorla et al., 2002, 2008; Gutiérrez-Barranquero et al., 2013b). In addition, several studies have attempted to unravel the biosynthesis pathway and the regulatory mechanisms of mangotoxin production (Arrebola et al., 2007; Arrebola et al., 2012; Carrión et al., 2012, 2014). A molecular evolutionary approach using mangotoxin biosynthetic operon gene cluster, revealed that this operon was specifically distributed within the *P. syringae* Genomospecies 1, and which was acquired only once during evolution (Carrión et al., 2013). Moreover, a diversity survey of Pss strains isolated from mango trees was performed using phenotypic, genetic and phylogenetic approaches based on MLST analysis (Gutiérrez-Barranquero et al., 2013a) in order to understand the epidemiology of BAN disease. This study strongly indicated that Pss isolated from mango trees were forming a single phylotype inside the Pss species, characterized mainly by its adaptation to the mango host and by the production of mangotoxin. Subsequently, and due to the genome sequencing project of the model strain Pss UMAF0158 (Martínez-García et al., 2015), a gene cluster involved in the production of cellulose was discovered (Arrebola et al., 2015). This study demonstrated that cellulose was an important exopolysaccharide (EPS) to attach to the mango surface that could also act as a switch modulating the transition from epiphytic to pathogenic phases of Pss on the mango host. Finally, a PFPs sequencing project determined the importance of the 62-kb plasmids in improving the epiphytic survival of Pss strains isolated from mango trees (Gutiérrez-Barranquero et al., 2017a).

Therefore, this review summarizes the work that has been conducted on Pss strains isolated from mango trees over more than two decades of research. This phytopathogenic bacterium has arisen as a particular pathogen developing important features that modulate their epiphytic and pathogenic lifestyle phases on the mango tree surface.

## *Pseudomonas syringae* pv. *syringae*, the Causal Agent of Bacterial Apical Necrosis of Mango Trees

Mango crops (*Mangifera indica* L.) are present in many tropical and subtropical regions and represent one of the most important subtropical fruit crops distributed worldwide (Galán-Saúco, 2015). This crop was established in Southern Spain in Malaga in the early 1980s. The pace of the planting of this crop was relatively high over the last few years, expanding from 800 hectares (ha) in 2004 to 4500 ha in 2016 in Spain, of which more than 2,000 ha are in full production (Gutiérrez-Barranquero et al., 2017b). Very recent data claim that there are more than 6,000 ha, of which more than 3,000 are currently in full production, which would break the historical record of more than 30,000 tons of mango fruit harvested (Anonymous, 2018) August. Thus, the mango crop has been considered one of the most promising crops in Southern Spain, mainly in the tropical coastal areas of Malaga and Granada. As new crops are deployed in new regions, there might be spill-over effects and the emergence of new diseases. The commercial viability of this crop has been threatened by different bacterial and fungal plant pathogens (Bradbury, 1986; Gagnevin and Pruvost, 2001; Gutiérrez-Barranquero et al., 2019). In Southern Spain, the fungal pathogen *Fusarium mangiferae* which causes mango malformation disease (Crespo et al., 2012) and Pss the causal agent of BAN disease (Cazorla et al., 1998) are the most severe phytopathogens causing important economic losses. The main symptomatology associated with BAN disease, the isolation and identification of Pss as the causal agent of BAN disease, and the control methods specifically tested to limit and prevent Pss infections are discussed in detail below.

### BAN Disease Symptomatology

**FIGURE 1:**
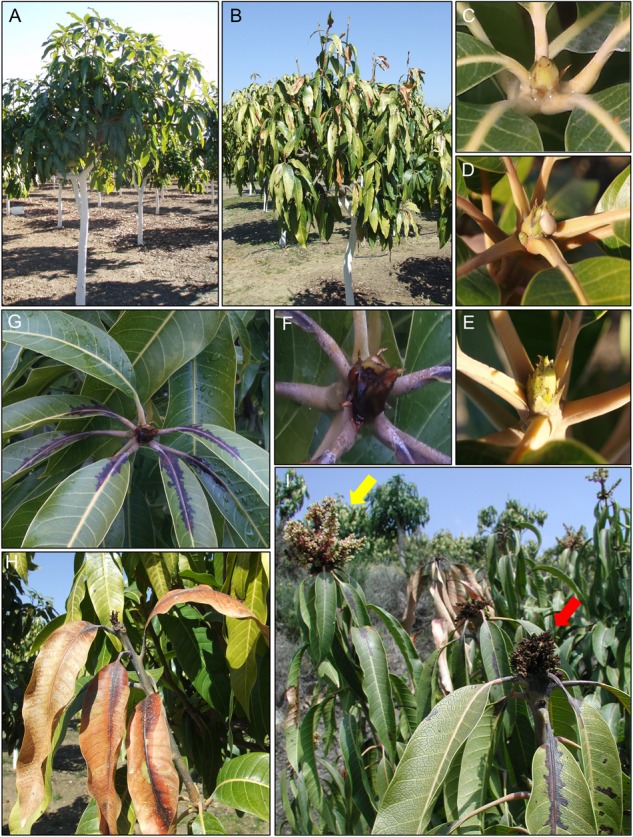
Typical symptoms of bacterial apical necrosis (BAN) disease on mango trees. **(A)** Healthy mango tree. **(B)** Mango tree affected by BAN disease. **(C)** Healthy mango apical bud. **(D)** Typical gum exudes on mango apical bud. **(E)** Initial necrotic spots on mango apical bud. **(F)** Severe necrosis of mango apical bud. **(G)** Necrotic symptoms progression from apical bud to leaves through the petiole. **(H)** Dead mango apical bud and surrounded leaves. **(I)** Flower panicles. Yellow arrow: healthy mango flower panicle; red arrow: necrosis on mango flower panicle.

The mango crop develops well at temperatures between 20 and 25°C, reaching a dormancy period when the temperature is below 15°C (Samson, 1986; Galán-Saúco, 2015). Thus, cool temperatures and wet periods play an important role in favoring the development of BAN symptoms, which has also been described in other infections caused by *P. syringae* in other woody hosts (Kennelly et al., 2007). Rain or dew are essential for inoculum dissemination to other buds and leaves, and wind exposure facilitates BAN development by causing microinjuries (Cazorla et al., 1998). BAN disease on mango trees is characterized by rapidly expanding necrotic spots on buds and leaves from October–November. January–February are the coolest and rainiest months in Southern Spain, giving rise to the highest incidence of necrotic symptoms, which is consistent with the period with the largest Pss population on mango trees (Cazorla et al., 1998). Additionally, at this time the symptoms can extend from buds through the leaf petiole to reach the leaves and stems. Typically, lesions on leaves start as interveinal, angular, water-soaked spots that may coalesce, becoming black and slightly raised. Importantly, favorable weather conditions for the pathogen that are maintained throughout the winter and even into the spring season can promote the appearance of wood necrosis on branches to such a degree that, in extreme cases, this can lead to the death of the tree. These symptoms are quite similar to those described for blossom blast of pear and stone fruits (English et al., 1980). Additionally, a white milky gum exudate can also be observed. Necrotic symptoms affecting flower panicles are less frequently observed but can become very apparent in years with severe attacks. These symptoms cause the most severe economic losses due to decreases in fruit yield (Cazorla et al., 1998). The typical symptoms of BAN disease of mango trees are summarized in Figure 1.

### Unraveling the Causative Agent of BAN Disease

The phytopathogenic bacterium *P. syringae* has the ability to survive as an epiphyte on plant surfaces. During its epiphytic phase, *P. syringae* has to cope with different abiotic factors by using different mechanisms (Sundin and Jacobs, 1999; Yu et al., 1999; Lindow and Brandl, 2003), which allow it to achieve large population sizes before starting an infection process (Hirano and Upper, 2000). Although *P. syringae* can elicit disease symptoms in a wide variety of woody and herbaceous plants, *P. syringae* has been considered a weak pathogen because the infection process on their plant hosts can be strongly improved by frost damage or mechanical injury. Thus, *P. syringae* can elicit disease outbreaks in temperate regions distributed worldwide in important crops, causing significant yield losses (Kennelly et al., 2007). Since the early 1990s, necrotic symptoms have been observed in apical buds, leaves and stems in mango trees in Southern Spain and Portugal (Cazorla et al., 1998). In years with severe attacks, which correlate with cool and wet winters, necrotic symptoms were more evident in the mango tree canopy and could cause a reduction of 30–50% in mango fruit production (Gutiérrez-Barranquero et al., 2012). Preliminary isolation from the edge of necrotic tissues of mango trees revealed that over 90% of bacterial isolates recovered were fluorescent *Pseudomonas*. Similar necrotic symptoms have been reported in many other woody hosts infected by Pss, such as peaches (Endert and Ritchie, 1984), citrus (Mirik et al., 2005; Ivanović et al., 2017), cherry (Sundin et al., 1989; Wenneker et al., 2013), almond (Lindow and Connell, 1984), apple (Mansvelt and Hattingh, 1986; Gasic et al., 2018) and pear (Montesinos and Vilardell, 1991; Xu et al., 2008). Different biochemical and physiological characteristics suggested the tentative identification of *P. syringae*. Furthermore, the presence of ice nucleation activity (INA), a virulence trait well-documented in *P. syringae* to be used by the bacterium to cause micro-wounds on the plant surface to provide an entry way to the plant (Hirano and Upper, 1995; Hwang et al., 2005), was found in bacterial isolates following a protocol previously described by Cazorla et al. (1995). The production of lipodepsipeptidic toxins typically associated with *P. syringae*, such as syringomycin and syringopeptins, were also confirmed in bacterial isolates from mango (Gross and DeVay, 1977; Ballio et al., 1991; Arrebola et al., 2003). All the results obtained conclusively confirmed that the bacterial isolates associated with necrotic symptoms in mango trees belonged to the *P. syringae* species (Cazorla et al., 1992, 1998). *P. syringae* is a highly heterogeneous species comprising more than 60 pathovars (Young, 2010). To determine which pathovar was the causal agent of necrotic symptoms, different pathogenicity tests were performed in tomato and lilac plants, immature lemon and pear fruits, and bean pods (Lelliott and Stead, 1987). All *P. syringae* strains assayed induced typical symptoms in all plant hosts of Pss. Once the bacterial strains associated with necrotic symptoms in mango trees were identified, a pathogenicity test in adult mango plants was carried out in order to fulfill Koch’s postulates. Two different experiments under field conditions were performed using 2-year-old mango plants growing in pots. Buds and stems were inoculated with 10 μl of bacterial suspensions using a microsyringe. Necrotic symptoms developed in the inoculated mango trees, and the incidence and severity of necrotic symptoms that occurred in each experiment (i.e., different years) were different, indicating the importance of the weather conditions in symptom development, as has been previously observed for *P. syringae* in other hosts (Hirano and Upper, 2000). The subsequent re-isolation from the necrotic lesions artificially reproduced in mango tissues and the subsequent identification confirmed that Pss was the causal agent of bacterial apical necrosis (BAN) of mangos (Cazorla et al., 1998).

**FIGURE 2:**
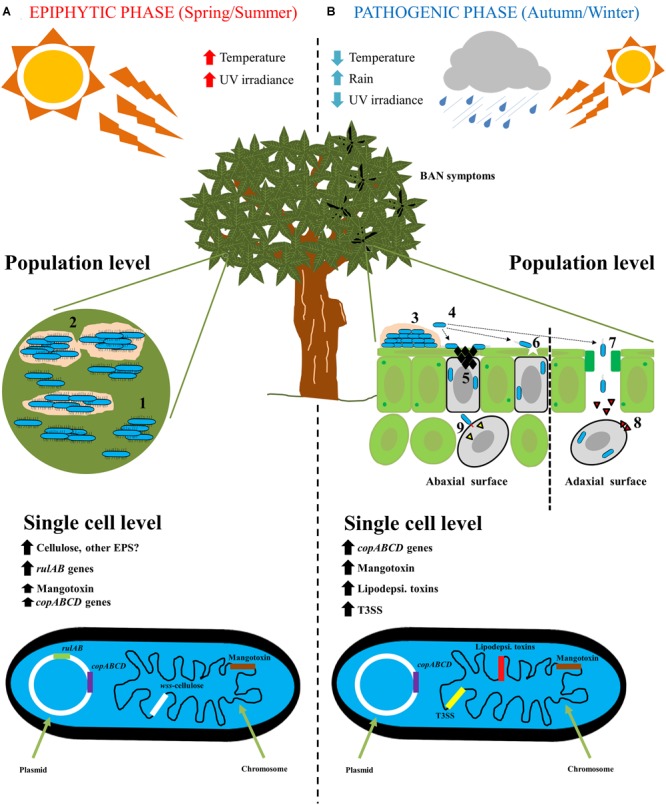
The life cycle of *Pseudomonas syringae* pv. *syringae* on mango trees. **(A)** Epiphytic phase of *P. syringae* pv. *syringae* on mango trees is developing mainly in spring/summer seasons, where high temperature and high UV radiation are present. At population level, *P. syringae* pv. *syringae* is present mainly on the buds and leaves surfaces forming microcolonies (1), that will subsequently form a mature biofilm with the biosynthesis of an extracellular matrix (2). At single cell level, *rulAB* operon encoded by 62-kb PFP plasmid involved in resistance to UV radiation, and *wss* operon present at the chromosome and involved in the biosynthesis of cellulose, are both highly expressed. On the contrary, *copABCD* operon encoded by 62-kb PFP plasmid involved in copper resistance, and *mbo* operon located at the chromosome and involved in mangotoxin biosynthesis, are less expressed. **(B)** Pathogenic phase of *P. syringae* pv. *syringae* on mango trees arise primarily in autumn/winter seasons, where low temperatures, low UV radiation and high rainfall are present. At population level, the infection process on mango leaves and buds is the following: (3) epiphytic survival and biofilm formation; (4) biofilm disassembly and bacterial migration; (5) Ice nucleation activity to damage mango surfaces; (6) bacterial entry into cells by microinjuries; (7) Bacterial entry into cells through stomata; (8) Release of phytotoxins; and (9) Release of type III effectors by using the type III secretion system. At single cell level, firstly, *copABCD* operon involved in detoxification of copper compounds is highly expressed in response to copper treatment applications by farmers. Then, all genes that encode virulence factors are highly expressed (Mangotoxin, lipodepsipeptidic toxins and type III secretion system and its effectors) to elicit the typical BAN disease symptoms.

Therefore, the life cycle of Pss on mango trees is clearly divided first, in an epiphytic phase, in which Pss has to survive and grow under harsh environmental conditions, and second, in a pathogenic phase to produce BAN symptomatology. In both phases, different genetic traits are expressed to either, improve survival or to enhance an infection process (Figure 2).

### Control Options for BAN Disease

Management of woody plant diseases caused by *P. syringae*, and particularly those provoked by Pss, are a major concern for growers worldwide due to the broad host range. Sprays of copper compounds have been used for decades as standard bactericides to combat many bacterial diseases, but their use is subject to a number of constraints (Kennelly et al., 2007). The most common treatment for controlling BAN disease in Southern Spain is the spraying of a copper compound with a film-forming mode of action known as Bordeaux mixture (BM). However, different copper-based compounds fail to protect against BAN. Unfortunately, continuous treatments with copper sprays can lead to many problems. The efficacy of copper treatments for the control of bacterial diseases is often limited, largely due to the selection of copper-resistant strains; this has previously been described for Pss strains isolated from mango trees (Cazorla et al., 2002). Another serious problem associated with the excessive usage of copper is that copper is a major heavy metal contaminant that accumulates in soil from different sources (Wang, 1997; Xiong, 1998; Kabata-Pendias, 2001). Copper has demonstrated toxicity to roots and young shoots and leaves (Kairu et al., 1985; Alva and Graham, 1991; Iannotta et al., 2007), and has sustained bioaccumulation effects (Xiong and Wang, 2005). Finally, the European Union has introduced legislation limiting the use of copper compounds in regulation No. 473/2002 (Anonymous, 2002). For all of these reasons, there has been an urgent need expressed by growers and extension services to search for alternative treatments to copper compounds that may be effective for the control of BAN disease. In this context, Cazorla et al. (2006) evaluated the capacity of several different control treatments to cope with BAN disease in mango crops. In addition, the mechanisms of action of the different treatments were examined, analyzing their effect on Pss population levels. The treatments assayed in this work included BM, fosetyl-Al, gibberellic acid, acibenzolar-*S*-methyl, silicon gel (soluble potassium silicate 34%) and combined treatments (Cazorla et al., 2006). Interestingly, treatments reduced symptoms but did not reduce the size of the pathogen population, suggesting a non-bactericidal mode of action of these compounds. After evaluation of the different treatments, this study concluded that the best treatment to control BAN disease was conventional copper-based treatment BM. However, there were promising effects showed by other assayed treatments against BAN disease, indicating that a few of them could be interesting alternatives to traditional chemical control (Cazorla et al., 2006). The silicon gel was highly relevant, because its reduction of necrotic symptoms in apical buds was similar to the levels obtained with BM; it also has potential for use in organic farming.

Due to the limitations concerning the use of copper compounds, together with the increasing demand for organic crops, have led to in-depth analysis of different alternative treatments to combat plant diseases. Particularly, Gutiérrez-Barranquero et al. (2012) performed a study where they analyzed different alternative treatments, including the silicon gel that previously showed potential to control BAN disease. In this study after different scale trials (small, semi-commercial, and commercial), confirmed the efficacy of silicon gel to control BAN disease, reducing the occurrence of necrotic symptoms at a similar level to the conventional treatment BM. Moreover, mango growers directly observed the effectiveness of silicon gel, and thus, this treatment has been registered for commercial use in mango crops in Spain as a phytostrengthener compatible with organic farming (Gutiérrez-Barranquero et al., 2012). Interestingly, silicon gel failed to reduce the bacterial population in mango tress, suggesting a film-forming mode of action acting as a physical barrier to avoid the entry of the pathogen, as it was previously reported for BM (Becerra, 1995). A similar mode of action has been previously described for silicon protective effects in other plant hosts against fungal and bacterial pathogens (Diogo and Wydra, 2007; Guével et al., 2007; Sun et al., 2010). However, other putative modes of action for silicon gel cannot be ruled out, as might be the induction of systemic resistance (ISR) (Bélanger et al., 2003; Rodrigues et al., 2003; Rodgers-Gray and Shaw, 2004; Fauteux et al., 2005) and to enhance cell wall lignification (Kim et al., 2002).

## Epiphytic Fitness Determinants: Improving Survival of *P. syringae* pv. *syringae* on Mango Surfaces

Plant surfaces are hostile and dynamic environments for plant-associated bacteria due to rapidly changing climatic conditions (Lindow and Brandl, 2003). *P. syringae* is an epiphytic bacterium and an opportunistic plant pathogen that needs to survive on plant surfaces (Hirano and Upper, 2000). Before initiating infection, *P. syringae* has to face environmental abiotic stressors via different survival mechanisms (Sundin and Jacobs, 1999; Yu et al., 1999; Lindow and Brandl, 2003). The life cycle of Pss on mango plant surfaces (as depicted in Figure 2) involves an epiphytic phase mainly during the spring and summer seasons, that subsequently leads to an infection process during the autumn and winter seasons, when the weather conditions are favorable for the disease development (Cazorla et al., 1998).

*Pseudomonas syringae* pv. *syringae* isolated from mango trees has therefore developed different strategies to survive on the mango plant surface. Where present, the 62 Kb PFP plasmids exhibit a key role (Cazorla et al., 2002, 2008; Arrebola et al., 2009; Gutiérrez-Barranquero et al., 2013b, 2017a). Recently, other important genes located on the chromosomal genetic material have been described as having a primary role in adhesion and subsequent biofilm formation on mango plant surfaces (Arrebola et al., 2015).

### Copper and Ultraviolet Resistance Genes Mainly Encoded by PFP Plasmids Are Essential for Epiphytic Survival on Mango Tree Surfaces

Plasmids have been reported to be one of the most important sources for bacterial evolution, due to their ability to acquire foreign DNA and be rapidly transmitted among bacteria via the horizontal gene transfer process (Vivian et al., 2001; Norman et al., 2009). Plasmids are part of the flexible genome and represent a portion of the genome that does not contribute to basic survival functions. However, plasmids encompass important genes that can improve the ecological fitness of their bacterial hosts (Medini et al., 2005; Sundin, 2007) and improve virulence mechanisms (Jackson et al., 1999; Arnold et al., 2001). The PFPs are a family of native plasmids that appear to be indigenous to *P. syringae*. All PFP plasmids share a major replication protein, gene *repA* (Sesma et al., 1998, 2000). Apart from specific genes involved in self-maintenance and replication processes of PFPs, different genes implicated in virulence and/or ecological fitness are encoded. In particular, copper- and ultraviolet radiation-resistance genes are two of the most widely distributed genes in this family of plasmids, which play a fundamental role in epiphytic survival (Sundin, 2007).

As mentioned previously, the use of copper compounds has been strongly associated with agriculture (Lamichhane et al., 2018). The extensive use of copper by growers led to an increase in the dosage and frequency of applications, giving rise the emergence of copper-resistant strains, a concerning issue that is very common among plant pathogenic bacteria, such as *P. syringae* (Sundin et al., 1989; Andersen et al., 1991; Sundin and Bender, 1993; Scheck and Pscheidt, 1998). In Southern Spain, different copper compounds have been largely used to control BAN disease in mango trees, as well as other plant diseases. This suggests that the selection of copper-resistant strains could be a major reason for further control failures with copper bactericides. The *copABCD* operon is the most common genetic determinant associated with copper resistance in *P. syringae* and has been reportedly associated with conjugative native PFP plasmids (Bender and Cooksey, 1986; Cooksey, 1987; Lim and Cooksey, 1993; Sundin and Bender, 1996). The *copABCD* operon encoded by a 35-kb plasmid from *P. syringae* pv. *tomato* was the first of these genes to be sequenced (Mellano and Cooksey, 1988). Based on this background, a study was performed to analyze the role of the *copABCD* operon in copper treatment tolerance, as well as its association with PFP plasmids in Pss strains isolated from mango trees (Cazorla et al., 2002). The presence of the *copABCD* operon and its association with PFPs plasmids was further analyzed. Over 75% of the copper-resistant strains, harbored 62-kb plasmids that showed a hybridization signal by Southern blot analysis with the *copABCD* probe obtained from *P. syringae* pv. *tomato* PT23 (Bender and Cooksey, 1987). The *copABCD* operon is also encoded, albeit to a lesser extent, in the chromosome, as well as in 120- and 45-kb plasmids. This observation suggested that different variants of copper resistance determinants could be found in Pss mango populations, as has previously been reported in other Pss populations (Sundin and Bender, 1993; Rogers et al., 1994). These data were also supported by 62-kb plasmids restriction profiles, identifying different restriction profiles in both copper-resistant and copper-sensitive plasmids. Moreover, in order to determine whether those plasmids were conjugative and also the main determinants of copper resistance, mating experiments proved that those plasmids were conjugative and were involved in the copper resistance phenotype. The presence of copper-resistant conjugative plasmids could be considered the main cause of control strategy failures when treating with copper bactericides. Thus, field experiments where copper treatments were applied to mango trees once per month, from September to June, were analyzed to assess the emergence of copper-resistant strains. It was clearly demonstrated that excessive usage of copper in mango trees to control BAN disease promoted an increase in copper-resistant strains, which could be mainly due to the ability of these plasmids with be transmitted by conjugative processes (Cazorla et al., 2002).

Subsequently, based on a PFPs sequencing project that included strains that harbored different variants of copper-resistance determinants (Gutiérrez-Barranquero et al., 2017a), it was shown that the presence of a novel genetic structure in Pss UMAF0081 strain isolated from mango increased copper-resistance phenotypes. This novel genetic structure encoded the c*usCBA* genes (detoxifying monovalent cations of silver and copper) and *copG*, a putative metal-transporting P-type ATPase, both inserted within the *copABCD* operon (Gutiérrez-Barranquero et al., 2013b). Furthermore, the novel genetic structure was found in another strain of Pss analyzed in this study (Pss 6–9 strain isolated from sweet cherry), and was also present in another two strains from the database that belonged to different pathovars (ATCC1128 pv. *tabaci* and NCPPB1108 pv. *tomato*). This structure encompassed 15 genes that were more than 17 kb in size, according to data that was recently updated (Gutiérrez-Barranquero et al., 2017a). To determine whether those extra genes were responsible for the increase in copper resistance, the minimal inhibitory concentrations of copper and other heavy metals were investigated. A collection of Pss strains isolated from mangos and others hosts, two strains from different pathovars, a transconjugant strain obtained previously (Cazorla et al., 2002, FF5-km + 62-kb 0081 plasmid), and two Pss FF5 transformants that harbored *copG* and *cusCBA* were independently evaluated. It was observed that the transconjugant strain showed the same MIC value for copper as the original 0081 strain; the transformed strains also had increased their MIC values in comparison with the copper-sensitive parental FF5 strain (Sundin and Bender, 1993). A growth curve performed in minimal medium supplemented with 0.8 mM of copper sulfate clearly demonstrated that *copG* and *cusCBA* were responsible for the increase in copper resistance. The role of *cusCBA* in detoxifying heavy metals has been previously reported in *Cupriavidus metallidurans* (Mergeay et al., 2003; Von Rozycki and Nies, 2009), *Escherichia coli* (Franke et al., 2003) and *Pseudomonas putida* KT2440 (Cánovas et al., 2003; Leedjärv et al., 2008). Finally, qRT-PCR experiments were performed to analyze the expression profiles of *copG* and *cusA* in the presence or absence of 0.8 mM copper sulfate. The results showed that the expression levels of *cusA* and *copG* increased 13- and 100-fold, respectively, in the presence of copper, and the expression of *cusA* was 3-fold higher than *copG*. These results confirmed the previous results obtained in the MIC and growth curve experiments, supporting the hypothesis that the novel rearrangement of three different genetic determinants into a conjugative plasmid increases copper resistance in *P. syringae* (Gutiérrez-Barranquero et al., 2013b). Thus, the presence of different copper-resistance structures associated primarily with 62-Kb PFPs plasmids has been demonstrated in Pss strains isolated from mango trees. However, little is known concerning the dynamics of maintenance or preference of the different types of 62-kb plasmids in Pss mango populations.

UV radiation affects bacterial communities that are intimately associated with plant surfaces; to overcome this growth-limiting environmental stress, different mechanisms have been developed (Beattie and Lindow, 1995; Sundin and Jacobs, 1999; Jacobs and Sundin, 2001). Among the different mechanisms described for avoiding UV damage, the presence of DNA repair mechanisms, such as *rulAB* operon encoded by PFP plasmids, are the most relevant in Pss (Sundin et al., 1996; Sesma et al., 1998; Sundin and Murillo, 1999; Sundin et al., 2000). In Southern Spain, mango crops are exposed to high UV radiation, especially in the spring and summer seasons. These highly restrictive solar radiation conditions suggest that a similar *rulAB*-like operon could play an indispensable role in the epiphytic survival of Pss associated with mango trees. As noted above, there was a high incidence of 62-kb plasmids associated with Pss isolated from mango trees that belong to the PFP family, which were also strongly associated with copper resistance phenotype (Cazorla et al., 2002). In this sense, Cazorla et al. (2008) analyzed the presence of the *rulAB*-like operon and its role in UV radiation tolerance in the 62-Kb PFP plasmids. Over 62% of the strains analyzed harbored a 62-kb plasmid. Additionally, it was observed that the Pss strains harboring 62-kb plasmids, rather than those lacking plasmids or having a different plasmid, were more tolerant to UVC exposure and were able to maintain higher population levels *in vitro*. However, the UVC wavelengths do not naturally reach the earth’s surface; thus, its impact on ecological fitness is low (Kim and Sundin, 2000). Subsequently, two different exposures conditions of UVA+B (high irradiation and similar radiation in a summer day in Southern Spain) were tested, and in both conditions, the role of plasmids in UVA+B tolerance was demonstrated. This result reinforced the importance of 62-kb plasmids in epiphytic survival of Pss isolated from mango trees in Southern Spain. Finally, the role of 62-kb plasmids in UV tolerance was tested *in vivo* on mango leaf surfaces, evaluating different conditions (leaves in sunny and shady areas, and adaxial and abaxial parts of the leaves). Once again, a greater surviving population of Pss was observed in the strains harboring 62-kb PFP plasmids, although this difference was only notable in the adaxial side of leaves exposed to direct sunlight radiation (Cazorla et al., 2008). Therefore, it has been clearly demonstrated the *rulAB*+ Pss strains shown an advantage regarding their epiphytical fitness, and thus this operon plays a relevant role in growth and dispersion of Pss on mango surfaces during its harsh epiphytic phase suffered in Southern Spain. This competitive advantage may be promoting the selection and the dispersion of these plasmids among the mango microbiome.

### Cellulose Production Modulates the Epiphytic and Pathogenic Lifestyle of *Pseudomonas syringae* pv. *syringae* on Mango Surfaces

Exopolysaccharides (EPS) have been reported to play essential roles in plant colonization and epiphytic survival of plant-associated bacteria (Pfeilmeier et al., 2016), including *P. syringae* (Yu et al., 1999). Different EPS have been associated with different functions of *P. syringae* during the epiphytic phase on the plant surface, as well as with its pathogenic lifestyle. Alginate is one of the most-studied EPS in *P. syringae*, and its involvement in osmotic stress tolerance, epiphytic survival, and virulence has been well-established (Yu et al., 1999; Freeman et al., 2013). Although the role of alginate and levan are not directly related to biofilm formation (Laue et al., 2006), their role in the initial stages of adhesion prior to biofilm development cannot be ignored (Yu et al., 1999). In addition, the putative role of levan as a nutrient source in mature biofilms, as well as its activity as a barrier blocking the recognition by the plant during pathogenesis, have been proposed (Kasapis et al., 1994; Laue et al., 2006). Cellulose is an important EPS that is well-documented in many bacterial species (Römling and Galperin, 2015). It is an integral part of extracellular matrix components of biofilms, mainly in environmental and pathogenic *Pseudomonas* (Ude et al., 2006; Römling et al., 2013). It is noteworthy that cellulose also exhibits major roles in the modulation of virulence mechanisms in both human and plant pathogenic bacteria (Römling et al., 2013). Based on a “genome mining” approach using the complete genome sequence of the model strain Pss UMAF0158 (Martínez-García et al., 2015), an orthologous gene cluster to the operon *wss* of *Pseudomonas fluorescens* SBW25 involved in cellulose biosynthesis was identified in the chromosome (Rainey and Travisano, 1998; Spiers et al., 2002). This gene cluster is organized as an operon, and encompasses 14,642 bp that encodes nine genes with putative functions associated with cellulose production and acetylation. Additionally, the evolutionary history of this gene cluster revealed that it was present in both pathogenic and non-pathogenic *Pseudomonas*. In addition, the flanking regions of the cellulose gene cluster were consistent between Pss UMAF0158 and other *P. syringae* cellulose-producing strains, suggesting an identical chromosome location.

Epiphytic colonization by *P. fluorescens* SBW25 and its survival on plant surfaces is primarily due to cellulose overproduction by the *wss* operon (Gal et al., 2003; Spiers et al., 2003). The role of cellulose in biofilm formation of *P. syringae* pv. *tomato* DC300 has also been shown (Pérez-Mendoza et al., 2014). To determine the role of cellulose in the lifecycle of Pss isolated from mangos, insertional mutants in the biosynthetic genes of the *wss* cluster, *wssB* and *wssE* (Römling, 2002), were constructed and proved to be impaired in cellulose production. Furthermore, a cellulose-overproducing strain was obtained via the transformation of Pss UMAF0158 with plasmid pVS61-WsR19 that contained *wspR19* from *P. fluorescens* SBW25 (Ude et al., 2006). Scanning electronic microscopy on mango buds and tomato leaves revealed the formation of microcolonies of the wild-type and overproducing strains immersed in the extracellular matrix, but not for the cellulose-defective mutants. Furthermore, adhesion experiments on mango leaves revealed that the amount of bacteria recovered were higher in the wild-type and overproducing strains, in respect to the *wss* mutants. In contrast, although growth curves on minimal medium for the different strains exhibited similar patterns, the incidence (number of necrotic points developed) and the severity (necrotic area developed) on tomato leaflets were higher in the *wss* mutants, lower in the wild-type, and practically abolished in the cellulose overproducing strain. The competitive index approach analysis supported these results, showing that the competitiveness of the overproducing strain was decreased during the plant infection experiments (Arrebola et al., 2015). It is evident that cellulose plays a primary dual role between epiphytic and pathogenic lifestyle of Pss on mango tree surfaces, which suggests that this trait is maintained on Pss mango populations for mango leaf and bud colonization and adaptation. Mechanisms of the regulation of cellulose biosynthesis by Pss isolated from mango trees has not yet been determined, but some clues have been discovered in related *Pseudomonas*. The second messenger c-di-GMP controls cellulose biosynthesis in *P. fluorescens* SBW25 (Spiers et al., 2002) and regulates the switch between the static and motile phases in many different bacterial species (Römling et al., 2013). More recently, the transcriptional regulator AmrZ has been reported to be a key regulator in the biosynthesis of cellulose in *P. syringae* pv. *tomato* DC3000 (Prada-Ramírez et al., 2016). The regulon of the AmrZ transcriptional regulator includes putative c-di-GMP proteins such as AdcA and MorA; thus, AmrZ could be directly involved in cellulose biosynthesis by modulating the available pool of c-di-GMP.

## Virulence Factors Associated With *Pseudomonas syringae* pv. *syringae* Strains Isolated From Mango Trees

As described by Salmond (1994), a virulence factor could be any molecule present on the bacterial cell surface or released from the cell that could influence the growth of the pathogen in plants, enhancing infection and subsequent disease development. Plant pathogenic bacteria have developed many different and specific virulence strategies to infect successfully their plant hosts. The identification, characterization and dissection of the modes of action of different virulence factors is complex, despite the efforts of many research groups (Mansfield et al., 2012; Pfeilmeier et al., 2016). Whereas the traits that confer *P. syringae* pathogenicity are numerous and well-studied, the mechanisms underlying susceptibility of mango are unknown. The lack of balance in our understanding of the mechanisms involved (well understood for the pathogen, poorly understood for the host) make us to focus in the role of the pathogen during the interaction with the host. *P. syringae*, in particular, and Pss strains isolated from mango specifically, shows a broad and sophisticated armament of different virulence factors (Ichinose et al., 2013), among which bacterial toxins are one of the most studied in depth.

### Bacterial Toxins

Bacterial toxins are important virulence factors of *P. syringae* (Mitchell, 1991) and have been described to be involved in the development of chlorotic and necrotic disease symptoms in its plant hosts (Volksch and Weingart, 1998; Scholz-Schroeder et al., 2001). Lipodepsipeptidic toxins, such as syringomycins and syringopeptins, have been strongly associated with several pathovars of *P. syringae* and are mainly related with the production of necrotic symptoms (Gross and DeVay, 1977; Ballio et al., 1991; Adetuyi et al., 1995; Vassilev et al., 1996; Bender et al., 1999; Scholz-Schroeder et al., 2001). Pss strains isolated from mango trees were found to produce syringomycin by using growth inhibition tests toward *Geotrichum candidum* (Gross and DeVay, 1977) and *Rhodotorula pilimanae* (Iacobellis et al., 1992), and the detection of a specific gene involved in its biosynthesis was done by a PCR protocol (Sorensen et al., 1998). In addition, Pss strains isolated from mango trees were also found to produce syringopeptins by using a grown inhibition bioassay of *Bacillus megaterium* (Lavermicocca et al., 1997). Another group of important toxins described in several pathovars of *P. syringae* are the so-called “antimetabolite toxins.” This group of toxins blocks the function of enzymes involved in the biosynthetic pathways of crucial amino acids, as well as the biosynthesis of polyamine (Bender et al., 1999; Arrebola et al., 2011a,b). These toxins produce chlorotic symptoms in plant tissue due to the accumulation of different intermediates (Patil et al., 1972; Turner and Debbage, 1982; Bachmann et al., 1998). The best-known antimetabolite toxins produced by different pathovars of *P. syringae* are tabtoxin, phaseolotoxin, and the recently identified mangotoxin (Arrebola et al., 2003). Mangotoxin was initially identified to be produced mainly by Pss strains isolated from mango trees, although its production was also reported in a few Pss strains from other hosts (Arrebola et al., 2003). The biosynthesis pathway of mangotoxin, its regulation, and the role that this toxin plays in the different lifestyles of Pss-mango interactions are discussed extensively in the next section.

## Mangotoxin, an Antimetabolite Toxin Mainly Associated With *P. syringae* pv. *syringae* Strains Isolated From Mango Trees

**FIGURE 3:**
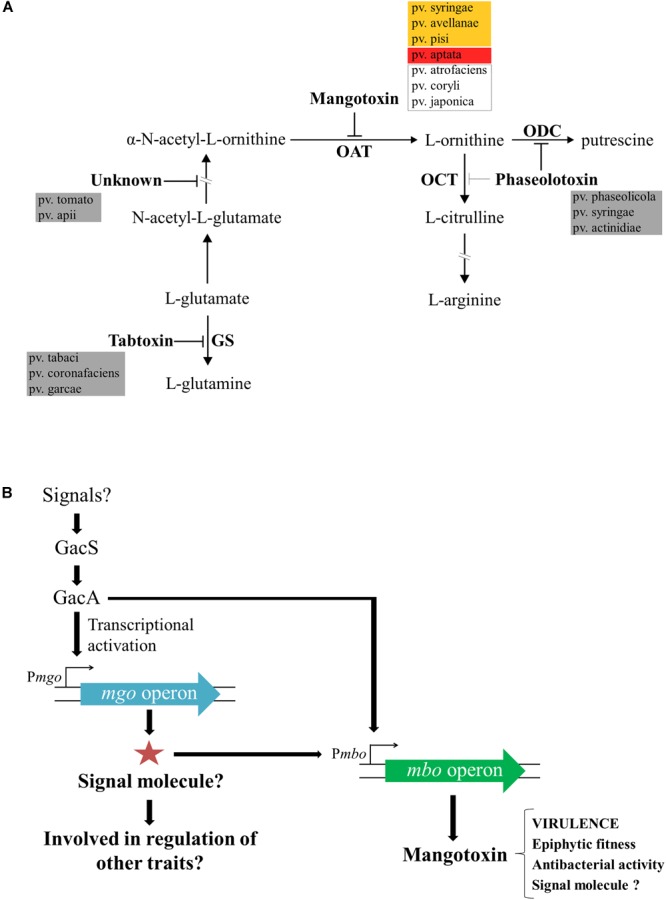
Enzymatic target of mangotoxin and its biosynthesis regulation. **(A)** Representative scheme of the arginine-glutamine and polyamine biosynthesis pathways. The enzymatic targets of the different antimetabolite toxins, including mangotoxin, and the different pathovars involved in the production of the different antimetabolite toxins are depicted. OAT, ornithine *N*-acetyltransferase; OCT, ornithine carbamoyltransferase; ODC, ornithine decarboxylase, and GS, glutamine synthetase. Orange box: pathovars positives for the presence of *mbo* genes and positives for mangotoxin production; red box: pathovar positive for the presence of *mbo* genes, but negative for mangotoxin production; dotted lines box: pathovars positive for the presence of *mbo* genes, but not experimentally tested for mangotoxin production. **(B)** Mangotoxin biosynthesis regulation model. GacS/GacA two-component regulatory system regulates directly or indirectly the transcription of the *mgo* operon. *Mgo* operon could synthetize a positive regulator (signal molecule) to activate the *mbo* operon transcription. The *mbo* operon produces mangotoxin, which acts primarily as a virulence factor, although, other functions have been described.

Mangotoxin is the most recent antimetabolite toxin discovered and was first described to be mainly produced by Pss strains isolated from mango trees. This toxin was called “mangotoxin” due to the plant host (mango tree) from which most of the Pss strains mangotoxin producers were isolated (Arrebola et al., 2003). As mentioned above, antimetabolite toxins block enzymes functions involved in the biosynthetic pathways of crucial amino acids and the biosynthesis of polyamine (Bender et al., 1999; Arrebola et al., 2011a,b). The toxic activity of mangotoxin is reversed by the addition of ornithine, and thus, its target enzyme was identified as ornithine *N*-acetyl transferase (OAT) (Arrebola et al., 2003). In Figure 3A, a schematic representation of the arginine-glutamine and polyamine biosynthesis pathways shows the target enzymes of the different antimetabolite toxins including mangotoxin. In order to decipher the chemical structure of mangotoxin a physicochemical characterization was performed initially using cell-free filtrates revealing that mangotoxin is a small secreted molecule of a hydrophilic nature smaller than 3 kDa in size, extremely resistant to high pH and high temperature, but sensitive to protease treatments. The analysis of a Tn5 defective mutant in mangotoxin production (UMAF0158-3aE10) and the wild-type strain Pss UMAF0158 by using High-performance liquid chromatography (HPLC), revealed a specific peak associated with mangotoxin activity (Arrebola et al., 2003). Another chemical separation techniques such hydrophilic interaction liquid chromatography (HILIC) and ion Exchange chromatography (FPLC) have been also applied to decode the mangotoxin structure (data not published). However, the efforts conducted to unravel the chemical structure of mangotoxin have been in vain to date, largely due to its high chemical instability.

To understand the molecular basis of mangotoxin production, three mutants impaired in mangotoxin production obtained from a genomic library (Pss UMAF0158-3γH1, -6γF6, and -5αC5) that displayed growth characteristics and production of lipodepsipeptidic toxins similar to wild-type strain UMAF0158 (Arrebola et al., 2007) were studied in depth. The insertion in the mutant UMAF0158-6γF6 was located in a DNA region that showed high similarity with an non-ribosomal peptide synthetase (NRPS) present in Pss B728a, *P. syringae* pv. *tomato* DC3000 and *P. syringae* pv. *phaseolicola* 1448A. This orf called *mgoA* gene has a size of 3447 bp, and the amino acids sequence of this protein was composed of an activation module with conserved domains typical for NRPS (Stein and Vater, 1996; Marahiel et al., 1997). The role of *mgoA* in virulence of Pss was demonstrated in tomato leaflets, showing this mutant a lower disease incidence than the wild-type. Therefore, the NRPS gene *mgoA* was confirmed to be involved in mangotoxin biosynthesis and, also, in virulence (Arrebola et al., 2007). Furthermore, three additional genes were detected together with the *mgoA* gene and were designated *mgoB, mgoC, mgoA*, and *mgoD*, in accordance with the mangotoxin generating operon. Insertional mutants in *mgoC, mgoA*, and *mgoD*, had altered mangotoxin production. Additionally, by using RT-PCR all *mgo* genes were co-transcribed together, forming a single polycistronic mRNA and thus forming an operon. Complementation experiments with the *mgo* operon restored the ability of the mutants to produce mangotoxin, and therefore, these results confirmed strongly that the *mgo* operon was necessary for mangotoxin production (Arrebola et al., 2007, 2012). The *mgo* operon has been found to be well-distributed in the majority of *Pseudomonas* species, including different pathovars of *P. syringae* (Lindeberg et al., 2008; Vallet-Gely et al., 2010). A homologous gene cluster to the *mgo* operon, *pvf*, has been proposed to be encoded in *Pseudomonas entomophila* as a regulator of virulence factors (Vallet-Gely et al., 2010). Recently, the family of pyrazine *N*-oxides (PNOs), including a novel dihydropyrazine *N,N*′-dioxide metabolite, were identified to be produced by the *pvf* gene cluster in *P. entomophila*, suggesting that these molecules could be involved in *Pseudomonas* signaling and virulence (Kretsch et al., 2018). In addition, fragin biosynthesis, the main antifungal compound produced by *Burkholderia cenocepacia* H111 is under the control of valdiazen, a novel quorum-sensing signaling molecule produced by a gene cluster homologous to the *mgo* and *pvf* operons (Jenul et al., 2018). Although the structure of the putative signaling molecule produced by the *mgo* operon in Pss isolated from mango trees remains unknown, its function as a regulator of biosynthesis of mangotoxin, and likely other secondary metabolites, is quite feasible.

Interestingly, another two Tn5 mutants abolished in mangotoxin production (UMAF01585aC5 and UMAF0158-4βA2), and thus, affected in virulence (tested in virulence assay in tomato leaflets) were studied in depth because they did not show homology with the genome sequences of Pss B728a, *P. syringae* pv. *tomato* DC3000 or *P. syringae* pv. *phaseolicola* 1448A. The involvement of mangotoxin in the epiphytic survival of Pss strains isolated from mango was demonstrated by Arrebola et al. (2009). Epiphytic survival experiments on tomato leaflets revealed that there was no difference between the wild-type Pss UMAF0158 and both mutants. Nevertheless, when the bacteria were co-inoculated together the wild-type with each of the mutants individually a slight but significant decrease was observed in the mutants, and the difference reached almost one order of magnitude. Thus, in addition to its virulence function, mangotoxin could also play a role in improving the ecological fitness of Pss strains isolated from mango trees. Furthermore, the screening of both mutant insertions in the genomic library showed that both were in a cluster of six genes present in wild-type strain Pss UMAF0158 and not in Pss B728a, *P. syringae* pv. *tomato* DC3000 or *P. syringae* pv. *phaseolicola* 1448A. Complementation experiments restored the ability of both mutants to produce mangotoxin (Carrión et al., 2012). These six genes were named *mboA, B, C, D, E*, and *F* in accordance with the mangotoxin biosynthetic operon and experiments based on RT-PCR and Northern blot analysis confirmed that these six genes were co-transcribed as a single polycistronic mRNA molecule confirming that these genes were forming an operon. Furthermore, site directed insertional mutations performed in each gene have shown a complete abolition of mangotoxin production in *mboA, B, C*, and *D* gene mutants and altered phenotypes in *mboE* and *F* gene mutants. Transformation experiments with pLAC-AF (pBBR1-MCS5 + mboA-F), a plasmid that contains the six *mbo* genes in different non-producing *Pseudomonas* strain genetic backgrounds, resulted in mangotoxin producers. Therefore, all experiments strongly confirmed that the *mbo* genes were essential for full production of mangotoxin.

Unambiguously, Carrión et al. (2014) demonstrated that the regulation of mangotoxin production was under the control of both *gacS*/*gacA* and *mgo* genes and additionally, that *mgo* genes were regulated by *gacS*/*gacA* genes. Tn5 mutants that were all defective in mangotoxin production (*mgoA* mutant, *mboD* mutant, *mboB* mutant, *gacS* mutant, and *gacA* mutant) were used to unravel the regulation of the mangotoxin biosynthetic pathway. Transcriptional analysis by qRT-PCR showed that expression levels of the *mboA, C, and E* genes were significantly lower in the *gacA* and *mgoA* mutants than in the wild-type; however, the *mgo* and *mbo* mutants did not affect the transcription levels of the *gacS*/*gacA* genes. These results suggested that the *gacS*/*gacA* system controls the regulation of both *mgo* and *mbo* operons and downstream the *mgo* operon controlled the regulation of the *mbo* operon, and thus controlling the mangotoxin production. Promoter fusion experiments using the *mbo* promoter showed high levels of β-galactosidase activity in the wild-type, whereas the expression was significantly lower in *mgoA, gacA*, and *gacS* mutants, supporting the results obtained previously. Taken together, a model for the regulation of mangotoxin production has been proposed (Figure 3B) (Carrión et al., 2014). In this model, it is proposed that *mgo* molecules could serve as signaling molecules, as has been previously described in similar bacteria, and may be involved in the regulation of other virulence traits in Pss strains isolated from mango trees. Moreover, other functions in addition to virulence have been described for mangotoxin, and its putative role as a signaling molecule has been hypothesized.

**FIGURE 4:**
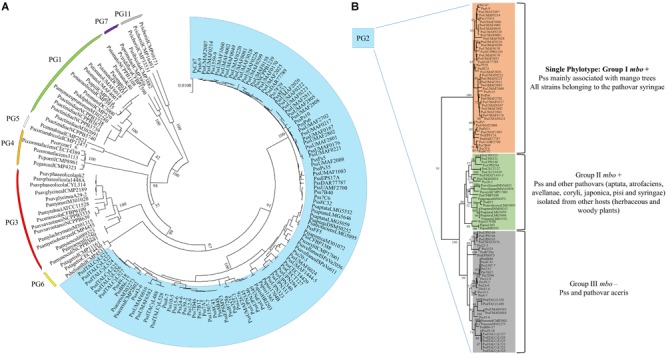
Multilocus sequence typing analysis of strains belonging to the *P. syringae* complex. **(A)** The neighbor-joining tree was constructed with combined partial sequences of *rpoD* and *gyrB* housekeeping genes using MEGA 7 software. Bootstrap values (1,000 repetitions) are shown on branches and evolutionary distances are in units of nucleotide substitutions per site. One hundred and fifty strains belonging to the phylogenetic groups 1, 2, 3, 4, 5, 6, 7, and 11 of the *P. syringae* complex are depicted in the circular phylogenetic tree. Marked in blue are represented the strains belonging to the phylogenetic group 2, where the *P. syringae* pv. *syringae* strains isolated from mango are found. **(B)** Exclusive representation of the phylogenetic group 2. Three main groups are defined regarding the presence or not of the *mbo* genes necessary for mangotoxin production. The topology was similar among phylogenetic trees produced by the maximum-parsimony and maximum-likelihood methods. Supplementary Table S1 provides the phylogenetic groups, the host of isolation and the accession numbers of the DNA sequences used for each strain represented in this phylogenetic analysis.

A diversity survey using different approaches (genetic, phenotypic, and phylogenetic) showed that Pss strains isolated from mango trees formed a single phylotype into the pathovar syringae associated with the mango host, producers of mangotoxin and distributed worldwide in areas where mango is grown and BAN is a relevant disease (Gutiérrez-Barranquero et al., 2013a). Despite of Pss strains isolated from mango are more similar among them in comparison with other Pss isolated from others hosts and other pathovars, phenotypic (including virulence degree) and genetic variability has been observed (Gutiérrez-Barranquero et al., 2013a). Then, in order to determine the evolutionary history of the *mbo* operon, a phylogenetic analysis using the housekeeping genes *rpoD* and *gyrB* grouped all strains belonging to the Genomospecies 1 together but separated in three different clusters. Two of these clusters were associated with the presence of the *mbo* operon (Carrión et al., 2013). Group I mbo+ was mainly composed of strains from the pathovar syringae, mainly isolated from woody hosts, but predominantly from mango trees, group which correspond with the single phylotype of Pss strains associated with mango trees described by Gutiérrez-Barranquero et al. (2013a). Group II mbo+ was composed of five different pathovars of *P. syringae* isolated from herbaceous and woody plants (aptata, avellanae, japonica, pisi, and syringae) and group III mainly composed by the pathovar syringae that was negative for the presence of *mbo* genes. Interestingly, group III (the group that lacked the *mbo* operon) diverged before the separation of groups I and II. These results suggested that the *mbo* operon was acquired by groups I and II in only one or two acquisition events after their separation from group III. Thus, this work strongly suggested that the *mbo* operon was horizontally acquired only once during the evolution of the *P. syringae* complex shown to be specifically distributed within the *P. syringae* Genomospecies 1 (Carrión et al., 2013). In the last few years, the databases have suffered a veritable explosion regarding the number of *P. syringae* genome sequences available (Baltrus et al., 2011; Thakur et al., 2016), which also contributed to a novel classification of the *P. syringae* complex in 13 different phylogroups (Berge et al., 2014). A more in depth phylogenetic analysis has been performed including 150 strains of the *P. syringae* complex belonging to the phylogenetic groups 1, 2, 3, 4, 5, 6, 7, and 11 (Figure 4). Inside the phylogenetic group 2, where the Pss strains isolated from mango are present, it is possible to observe the differentiation of three main groups, similar to those previously reported by Carrión et al. (2013). Group I mbo+ was mostly composed of pathovar syringae, mainly isolated from mango trees, corresponding with the single phylotype described. Group II mbo+ was composed of the 5 pathovars previously identified in this group. However, this new analysis included two more pathovars into this group (pathovar atrofaciens and coryli). Finally, a third group was composed mainly by the pathovar syringae that was negative for the presence of *mbo* genes. This new phylogenetic analysis confirms the previous assumption that Pss strains isolated from mango are forming a single phylotype inside the Genomospecies1-phylogenetic group 2.

### Ice Nucleation Activity

*Pseudomonas syringae* infections tend to be favored by cool and wet conditions due to its ability to induce ice nuclei formation at warm, subfreezing temperatures (-2 to -4°C) (Lindow et al., 1982; Hirano and Upper, 1995). Ice nucleation activity (INA), is considered an important virulence factor wide spread throughout *P. syringae* complex that plays a major role in the early stages of infections causing wounds that can facilitate disease particularly in woody plant species (Lindow et al., 1982; O’Brien and Lindow, 1988; Hwang et al., 2005; Lamichhane et al., 2014). In this sense, Cazorla et al. (1995) developed a simple and alternative multiple-tube test that showed an increase in detection sensitivity of active ice nuclei forming bacteria relative to the traditional drop-freezing methods (Lindow et al., 1978). This method revealed that all Pss strains isolated from mango trees were positive for INA detection. Although the INA virulence factor could be important at the initial stages of BAN development, the low probability of occurrence of frost in mango-producing areas makes its role in virulence largely anecdotal.

### Type III Secretion System

The most-studied and well-characterized virulence factor associated with *P. syringae* is the T3SS (Lindeberg et al., 2012). The T3SS is a complex nanomolecular machinery used by *P. syringae* and many other plant and animal pathogens to inject effector proteins into host plant cells to subvert the plant immune system and induce disease development (Lindeberg et al., 2012). While the T3SS is the most-studied virulence factor in *P. syringae*-plant interactions (Collmer et al., 2000; Oh et al., 2010; Cunnac et al., 2011; Lo et al., 2017), the role that this secretion system might play in the development of BAN disease has not been examined in depth to date. At this stage, a genome sequencing project performed on the Pss model strain isolated from mango trees, UMAF0158, revealed the presence of two different T3SSs (Martínez-García et al., 2015). The first T3SS (T3SS-1) is similar to the Hrp-1 T3SS family (Egan et al., 2014) found in different pathovars of *P. syringae* (Lindeberg et al., 2012) and represents the canonical T3SS widely distributed in pathogenic *P. syringae* strains (Block and Alfano, 2011; Lindeberg et al., 2012). Pss strains isolated from mango trees were able to induce a hypersensitivity response (HR) in tobacco plants (Cazorla et al., 1998). The capability of *P. syringae* to provoke a HR in non-host plants is dependent on a functional T3SS (Huang et al., 1992). Thus, in Pss UMAF0158, a simple deletion mutant constructed in the *hrpL* gene (UMAF0158Δ*hrpL*) (an alternative sigma factor that binds to the hrp box promoter sequence of the T3SS genes that upregulates their expression) confirmed the involvement of T3SS-1 in HR development (Martínez-García et al., 2015). The role of this particular T3SS in overall virulence has been widely recorded in Pss B728a, *P. syringae* pv. *tomato* DC3000, and many others (Schechter et al., 2006; Vinatzer et al., 2006; Kvitko et al., 2009; Lee et al., 2012).

Additionally, bioinformatics analysis highlighted the presence of an additional T3SS (called T3SS-2) in the chromosome of Pss UMAF0158 (Martínez-García et al., 2015) that was also found in different strains from different pathovars (Reinhardt et al., 2009; Studholme et al., 2009; Clarke et al., 2010; Matas et al., 2014). This T3SS-2 shows high similarity to the rhizobial-like T3SS Rhc of the Rhizobiales family (Gazi et al., 2012; Egan et al., 2014). A typical *hrp* box promoter regulatory sequences of the HrpL regulon found preceding the genes of the typical T3SS (Fouts et al., 2002) was missed in the T3SS-2. As it has been demonstrated in other *P. syringae* strains, the T3SS-2 is dispensable for pathogenicity, although a possible role in plant surface colonization or interaction with insects cannot be ruled out (Lindgren et al., 1986; Clarke et al., 2010; Pérez-Martínez et al., 2010; Silby et al., 2011). Different specific mutants in the T3SS-1, T3SS-2, and in combination in both systems constructed in Pss UMAF0158 did not revealed the function of the T3SS-2 in Pss isolated from mango trees, which remains unknown to date (Martínez-García et al., 2015).

**FIGURE 5:**
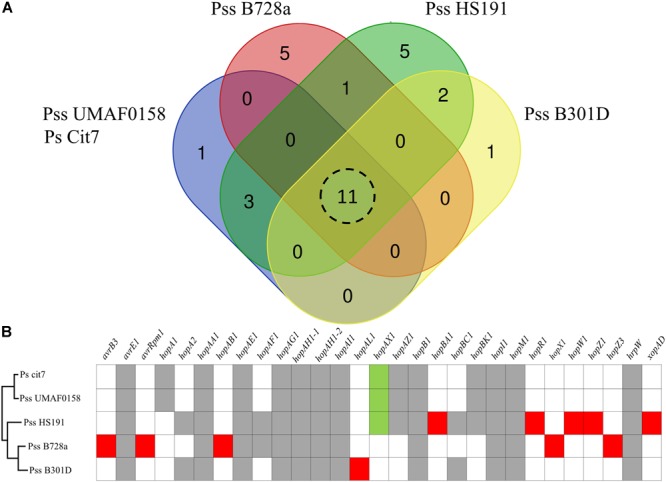
Type III effectors repertoire. **(A)** Venn diagram comparing the putative type III effectors presence in selected strains of *P. syringae* including the model strain *P. syringae* pv. *syringae* isolated from mango UMAF0158. Eleven type III effectors shared by all the strains analyzed. **(B)** Presence of specific type III effectors. Gray boxes indicate type III effectors presence in all the strains analyzed. Red boxes indicate type III effectors specific for each strain analyzed. Green boxes represents the hop*AX1*effector, an effector present in UMAF0158 that shows a high-specificity for a few strains of different pathovars exclusively belonging to the Genomospecies 1-Phylogenetic Group 2.

Due to the release of the Plant-bacteria Interaction FActors Resource (PIFAR), an open-access web-based resource for genetic factors involved in bacterial interactions with plant–hosts^1^ (Martínez-García et al., 2016), the detection of type 3 effectors (T3Es) has become more accurate than the method previously selected to identify T3Es in Pss UMAF0158 (Martínez-García et al., 2015). By using PIFAR tool, 15 putative T3Es have been identified in Pss UMAF0158, 4 T3Es more than the 11 previously identified. A Venn diagram analysis of the core T3Es, comparing the Pss UMAF0158 genome with the genome sequencing of three Pss strains (B728a, HS191, and B301D) and *P. syringae* Cit7 all belonging to Genomospecies 1 (Gardan et al., 1999) and Phylogenetic Group 2 (Berge et al., 2014), has been performed (Figure 5A). In addition, the presence or absence of the different T3Es present in these four strains are depicted (Figure 5B). *hopA1, hopAX1, hopAZ1*, and *hopBK1* are the unique T3Es shared by Pss UMAF0158 with several other strains (Ps Cit7 and Pss HS191). On the other hand, *hopA1, hopAX1*, and *hopBK1* have been found in other pathovars of *P. syringae* belonging to different phylogroups (Berge et al., 2014). Remarkably, the effector *hopAX1* appears to be mainly associated with a few strains of different pathovars all belonging to the Genomospecies 1-Phylogenetic Group 2 (pv. *aptata*, pv. *pisi*, pv. *aceris*, and pv. *syringae*). Dillion et al. (2019) have recently described the high-specificity of *hopAX1* T3E in the Phylogenetic Group 2.

## *P. syringae* pv. *syringae* Strains Isolated From Mango Trees in the Genomic Era

High-Throughput Sequencing technologies (HTS) has had a large impact on plant pathology and other research areas. In recent years, there has been substantial growth regarding genome sequencing of bacterial plant pathogens (Studholme et al., 2011) that can provide a strong basis for a better understanding of plant–microbe interactions that 1 day will contribute to the eradication of plant diseases. *P. syringae* is the model plant pathogen par excellence most often used worldwide to dissect plant–pathogen interactions (Baltrus et al., 2017). From the first genome sequenced of the model strain *P. syringae* pv. *tomato* DC 3000, the current landscape has changed markedly, with many groups interested in *P. syringae* comparative genomics and evolution (Lovell et al., 2009; Green et al., 2010; Baltrus et al., 2011; McCann et al., 2013; Thakur et al., 2016; Hulin et al., 2018). Currently, the complete genome sequences of 29 *P. syringae* strains, along with more than 400 draft genome sequences, are included in the NCBI database^2,3^. To date, there is only one complete genome sequenced of Pss strains isolated from mango trees (chromosome + 62-kb PFP plasmid), which was performed in the model strain Pss UMAF0158 (Martínez-García et al., 2015). This work revealed a high degree of conservation with other *Pseudomonas* from the *P. syringae* complex; however, different genetic factors were identified for their potential involvement in the epiphytic or pathogenic lifestyle, and these factors have been described in depth in this review. Among these factors, the most important were the presence of the *mbo* operon (mangotoxin biosynthetic operon), the presence of the *wss* operon (operon involved in cellulose biosynthesis), the additional type III-like rhizobial secretion system, the additional type VI secretion system, and a particular T3E repertoire.

Recently, a PFP sequencing project that includes 4 62-kb PFP plasmids from different strains of Pss strains isolated from mango trees was carried out (Gutiérrez-Barranquero et al., 2017a). In this work, it was revealed that the main functions of 62-kb plasmids of Pss strains isolated from mango trees were related to the increase in UV radiation and copper treatment tolerance. The backbone of the different plasmids regarding the genes involved in the maintenance, replication and conjugation was similar and showed a high degree of synteny. Interestingly, these plasmids were included in the previously described subgroup B (Ma et al., 2007), sharing more than the *repA* gene (replicase gene shared by all PFPs plasmids; Sundin, 2007). In addition, a novel genetic structure likely related to a cell-to-cell communication signaling system appeared in those plasmids upstream of the type IV secretion system, suggesting that the conjugation process could be under the regulation of this signaling mechanism (Gutiérrez-Barranquero et al., 2017a). On the other hand, there is a relatively low degree of homology in remaining genes found in each 62-kb PFP plasmids.

## Concluding Remarks and Future Directions

The enormous efforts that have been carried out over the last two decades have led us to gain more in-depth understanding of the *P. syringae* pv. *syringae*-mango host interactions. Pss causes important economic losses in mango crop production in the Mediterranean region. Pss strains isolated from mango trees form a single phylotype within the pathovar syringae and exhibit important factors that contribute to the epiphytic-pathogenic phase establishment on the mango plant, revealing a deep interaction between the pathogenic microbe and the host plant. It is worthy to note that the traits in *P. syringae* that are involved in pathogenic and epiphytic lifestyles have been studied in depth, but particularly, the mechanisms underlying the association of Pss with the mango host are little known. Thus, the major traits analyzed in depth in this review would help Pss to interact successfully with mango trees, but some of them are also useful in the interaction of other *P. syringae* strains with other plant hosts. Mangotoxin is the main virulence factor of this particular group of bacteria, and although much attention has been paid to it, the structure of this toxic molecule remains elusive. In addition, the possible role of mangotoxin as a signaling molecule modulating specific gene expression has been hypothesized. Further experiments are currently being carried out to confirm this hypothesis. Additionally, another important virulence factor not well-studied is the T3SS. In Pss isolated from mango trees, an extra copy of the T3SS is present. However, despite the efforts made, its role in the ecology of Pss remains unknown. Another relevant factor recently discovered in Pss strains isolated from mango trees is the presence of a cellulose biosynthetic gene cluster. The cellulose gene cluster has been described as involved in adhesion and biofilm formation development in Pss on the mango leaf surfaces. This gene cluster is only present in a few strains of *P. syringae* but is present in all Pss strains isolated from mango trees, suggesting that it is a crucial factor in the adaptation to the mango host. Its role in modulating epiphytic and pathogenic phases on mango surfaces has also been addressed. In addition, 62-kb PFP plasmids have been shown to play a key role in epiphytic survival of Pss on mangos, harboring UV and copper resistance determinants, among others. This long-lasting interaction among Pss and mango led us to search for effective control methods to allow farmers to deal with BAN symptoms. The efficacy of the alternative treatment silicon gel compared to the spray of copper compound BM has been demonstrated, and silicon gel has finally been registered for its commercial use in mango crops in Spain to combat BAN disease.

Given all of this, the future directions of this research are actually being targeted in two aims: (1) to unravel signaling mechanisms of Pss in interactions with other bacterial members of the mango microbiome by analysis of the transcript-level expression using *in vitro* and *in vivo* approaches; and (2) comparative genomics and evolutionary history analysis. In spite of the massive development of genomic sequencing technologies, there is a lack of information regarding genomic data from Pss strains isolated from mango trees. Thus, a great effort is currently being carried out to perform a major genome sequencing project involving a number of different strains to unravel the evolutionary processes that have occurred in mango populations from different geographical regions, separated in time. Phylogenetic and evolutionary approaches will open new windows of research that allow us to better understand why this phytopathogenic bacterium is so peculiar.

## Author Contributions

All authors listed have made a substantial, direct and intellectual contribution to the work, and approved it for publication.

## Conflict of Interest Statement

The authors declare that the research was conducted in the absence of any commercial or financial relationships that could be construed as a potential conflict of interest.

## Footnotes

1 http://bacterial-virulence-factors.cbgp.upm.es/PIFAR

2 https://www.ncbi.nlm.nih.gov/genome/185

3 https://www.ncbi.nlm.nih.gov/genome/2253
