# Supplementary material for: Pseudomonas syringae pv. syringae Associated With Mango Trees, a Particular Pathogen Within the “Hodgepodge” of the Pseudomonas syringae Complex
Source: Front Plant Sci. 2019 May 8;10:570. doi: 10.3389/fpls.2019.00570 (PMC6518948; doi:10.3389/fpls.2019.00570)
Supplement: Supplementary file 1 [file Data_Sheet_1.PDF]

Supplementary Table 1. Phylogenetic group, host of isolation and accession numbers of the different DNA sequences of each strain used for MLST analysis.

Bacterial strains	PG[a]	Host of isolation	*gyrB* partial sequence	*rpoD* partial sequence
P. amygdali pv.				
aesculi				
0893-23	3	Horse chesnut	NZ_AEAD00000000	NZ_AEAD00000000
2250	3	Chesnut	NZ_ACXT00000000	NZ_ACXT00000000
NCPPB3681	3	Chesnut	NZ_ACXS00000000	NZ_ACXS00000000
eriobotryae				
ICMP4455	3	Loquat	LJQI00000000	LJQI00000000
lachrymans	3			
M301315		Cucumber	NZ_AEAM00000000	NZ_AEAM00000000
mori				
MAFF301020	3	Mulberry	AEAG00000000	AEAG00000000
morsprunorum				
MAFF302280	1	Plum	NZ_AEAE00000000	NZ_AEAE00000000
tabaci				
ATCC11528	3	Tobacco	NZ_ACHU00000000	NZ_ACHU00000000
P. cannabina				
ICMP2823	5	Hemp	NZ_LJPX00000000	NZ_LJPX00000000
P. cichorii				
ICMP1649	11	Celery	NZ_RBPN00000000	NZ_RBPN00000000
ICMP3353	11	Tomato	NZ_RBRE00000000	NZ_RBRE00000000
ICMP6917	11	Safflower	NZ_RBRY00000000	NZ_RBRY00000000
P. coronafaciens pv.				
coronafaciens				
3113	4	Oat	NZ_RBUJ00000000	NZ_RBUJ00000000
CECT4389	4	Oat	JX867929	JX867851
garcae				
ICMP4323	4	Coffee	NZ_LJQK00000000	NZ_LJQK00000000
oryzae				
1_6	4	Rice	NZ_RBOG00000000	NZ_RBOG00000000
porri				
ICMP8961	4	Leek	NZ_LJRA00000000	NZ_LJRA00000000
P. syringae				
Cit7	2	Orange	AEAJ00000000	AEAJ00000000
P. syringae pv.				
aceris				
ICMP2802	2	*Acer* sp.	NZ_LJPM00000000	NZ_LJPM00000000
MAFF302273	2	Maple	NZ_AEAO00000000	NZ_AEAO00000000
actinidiae				
NCPPB3871	1	Kiwi	FR691735	FR691734
NCPPB3738	1	Kiwi	FR691737	FR691736
NCPPB3739	1	Kiwi	FR691739	FR691738
NCPBB3740	1	Kiwi	FR691741	FR691740
apii				
ICMP2814	1	Celery	NZ_LJPR00000000	NZ_LJPR00000000
aptata				
DSM50252	2	Sugar beet	AEAN00000000	AEAN00000000
LMG5059	2	Sugar beet	JX867932	JX867852
LMG5532	2	Sugar beet	JX867930	JX867853
LMG5646	2	Sugar beet	JX867931	JX867854
avellanae				
ISPaVe011	2	Hazelnut	EF203071	EF203069
ISPaVe2056	2	Hazelnut	EF203072	EF203070
atrofaciens				
LMG5095	2	Common wheat	NZ_JFZI00000000	NZ_JFZI00000000
cerasicola				
CFBP6109	3	Yoshino cherry	NZ_LT963391	NZ_LT963391
coriandricola				
ICMP12471	5	Coriander	NZ_LJPZ00000000	NZ_LJPZ00000000
coryli				
ICMP17001	2	Hazelnut	NZ_LJQC00000000	NZ_LJQC00000000
delphini				
ICMP529	1	*Delphinium* sp.	NZ_LJQH00000000	NZ_LJQH00000000
helianthi				

ICMP4531	6	Sunflower	NZ_LJQM00000000	NZ_LJQM00000000
japonica				
MAFF301072	2	Barley	AEAH00000000	AEAH00000000
macuolicola				
ICMP3935	1	Broccoli	NZ_LJQR00000000	NZ_LJQR00000000
pisi				
1704B	2	Pea	AEAI00000000	AEAI00000000
HRI203	2	Pea	NZ_QJTY00000000	NZ_QJTY00000000
NCPPB1365	2	Pea	JX867927	JX867849
primulae				
ICMP3956	7	*Primula* sp.	NZ_LJRC00000000	NZ_LJRC00000000
ribicola				
ICMP3882	7	Golden currant	NZ_LJRF00000000	NZ_LJRF00000000
tagetis				
CECT4430	6	Marigold	JX867928	JX867850
tomato				
DC3000	1	Tomato	NP_789866	NP_790384
DCT6D1	1	Tomato	JX867926	JX867848
K40	1	Tomato	NZ_ADFY00000000	NZ_ADFY00000000
NCPPB1108	1	Tomato	NZ_ADGA00000000	NZ_ADGA00000000
T1	1	Tomato	NZ_ABSM00000000	NZ_ABSM00000000
UMAF4002	1	Tomato	JX867867	JX867787
UMAF4007	1	Tomato	JX867924	JX867846
UMAF6018	1	Tomato	JX867925	JX867847
syringae				
7A7	2	Ornamental pear	JX867887	JX867843
7C6	2	Ornamental pear	JX867896	JX867815
7B12	2	Ornamental pear	JX867888	JX867807
7B40	2	Ornamental pear	JX867889	JX867808
7D46	2	Ornamental pear	JX867890	JX867809
7F29	2	Ornamental pear	JX867891	JX867810
8B48	2	Ornamental pear	JX867894	JX867813
8C32	2	Ornamental pear	JX867892	JX867811
8C43	2	Ornamental pear	JX867893	JX867812
8F21	2	Ornamental pear	JX867895	JX867814
5-10	2	Cherry	KC852127	KC852107
6-9	2	Cherry	JX867861	JX867782
8-7	2	Cherry	KC852128	KC852108
10-5	2	Cherry	KC852129	KC852109
12-9	2	Cherry	KC852130	KC852110
15-6	2	Cherry	KC852131	KC852111
20-3	2	Cherry	KC852132	KC852112
22-3	2	Cherry	KC852133	KC852113
24-6	2	Cherry	KC852134	KC852114
39-2	2	Cherry	KC852135	KC852115
43-6	2	Cherry	KC852136	KC852116
1444-5	2	Laurel	JX867917	JX867837
1507-7	2	Hawthorn	JX867871	JX867791
1559-9	2	Mango	JX867914	JX867834
4916	2	Bean	KC852138	KC852118
2676	2	Bean	JX867922	JX867842
3525	2	Bean	KC852137	KC852117
B728a	2	Bean	YP_233116	YP_237709
B86-17	2	Bean	KC852139	KC852119
CECT127	2	Lilac	JX867869	JX867789
CECT4429	2	Lilac	JX867870	JX867790
CFBP3388	2	Vetch	FR691733	FR691732
DAR77787	2	Mango	JX867911	JX867831
DAR77789	2	Mango	JX867912	JX867832
EPSMV3	2	Ornamental pear	JX867906	JX867826
EPS17A	2	Ornamental pear	JX867910	JX867830
FF5	2	Ornamental pear	NZ_GG700508.1	NZ_GG700503.1
ITACyL488	2	Vech	JX867876	JX867796
ITACyL522	2	Chickling pea	JX867883	JX867803
ITACyL523	2	Chickling pea	JX867882	JX867802
ITACyL524	2	Chickling pea	JX867881	JX867801
ITACyL525	2	Chickling pea	JX867880	JX867800
ITACyL526	2	Grass pea	JX867879	JX867799
ITACyL527	2	Grass pea	JX867878	JX867798
ITACyL528	2	Grass pea	JX867877	JX867797
NCPPB1239	2	Bean	JX867872	JX867792
Ps5	2	Mango	JX867873	JX867793
Ps6	2	Mango	JX867874	JX867794

Ps10	2	Mango	JX867875	JX867795
Ps35	2	Mango	JX867907	JX867827
UMAF0049	2	Mango	JX867913	JX867833
UMAF0081	2	Mango	JX867862	JX867783
UMAF0158	2	Mango	JX867863	JX867784
UMAF0167	2	Mango	JX867908	JX867828
UMAF0170	2	Mango	JX867909	JX867829
UMAF0176	2	Mango	JX867860	JX867781
UMAF0209	2	Mango	JX867916	JX867836
UMAF0214	2	Mango	JX867918	JX867838
UMAF0217	2	Mango	JX867899	JX867818
UMAF0220	2	Mango	JX867897	JX867816
UMAF0221	2	Mango	JX867900	JX867819
UMAF0222	2	Mango	JX867901	JX867820
UMAF0223	2	Mango	JX867902	JX867821
UMAF0225	2	Mango	JX867903	JX867822
UMAF0226	2	Mango	JX867898	JX867817
UMAF1003	2	Mango	JX867859	JX867780
UMAF1060	2	Mango	JX867858	JX867779
UMAF2007	2	Mango	JX867921	JX867841
UMAF2008	2	Mango	JX867864	JX867845
UMAF2025	2	Mango	JX867919	JX867839
UMAF2026	2	Mango	JX867920	JX867840
UMAF2700	2	Mango	JX867857	JX867778
UMAF2702	2	Mango	JX867856	JX867777
UMAF2801	2	Mango	JX867855	JX867823
UMAF2802	2	Mango	JX867904	JX867824
UMAF2805	2	Mango	JX867865	JX867785
UMAF2808	2	Mango	JX867905	JX867825
UMAF2811	2	Mango	JX867866	JX867786
UMAF3028	2	Mango	JX867915	JX867835
UMAF6016	2	Chestnut	JX867885	JX867805
UMAF6024	2	Purple phlomis	JX867868	JX867788
UMAF6582	2	Peach	JX867886	JX867806
UPN294	2	Bean	KC852140	KC852120
UPN323	2	Bean	KC852141	KC852121
UPN331	2	Bean	KC852142	KC852122
UPN340	2	Bean	KC852143	KC852123
UPN345	2	Bean	KC852144	KC852124
UPN346	2	Bean	KC852145	KC852125
UPN349	2	Bean	KC852146	KC852126
P. savastanoi pv.				
phaseolicola				
1448A	3	Bean	YP_272319	YP_272918
pk2	3	Kudzu	FR691745	FR691744
CYL314	3	Bean	FR691743	FR691742
savastanoi				
NCPPB3335	3	Olive	NZ_CP008742	NZ_CP008742
NCPPB639	3	Olive	AB039469	AB039514
glycinea				
A29-2	3	Soybean	NZ_AEGH00000000	NZ_AEGH00000000
ICMP2189	3	Soybean	NZ_LJQL00000000	NZ_LJQL00000000
P. viridiflava				
CFPB1590	7	Cherry	NZ_LT855380	NZ_LT855380

[a] PG: phylogenetic group
